# Positive Selection, Relaxation, and Acceleration in the Evolution of the Human and Chimp Genome

**DOI:** 10.1371/journal.pcbi.0020038

**Published:** 2006-04-28

**Authors:** Leonardo Arbiza, Joaquín Dopazo, Hernán Dopazo

**Affiliations:** 1 Pharmacogenomics and Comparative Genomics Unit, Centro de Investigación Príncipe Felipe (CIPF), Valencia, Spain; 2 Functional Genomics Unit, Bioinformatics Department, Centro de Investigación Príncipe Felipe (CIPF), Valencia, Spain; University of Texas, United States of America

## Abstract

For years evolutionary biologists have been interested in searching for the genetic bases underlying humanness. Recent efforts at a large or a complete genomic scale have been conducted to search for positively selected genes in human and in chimp. However, recently developed methods allowing for a more sensitive and controlled approach in the detection of positive selection can be employed. Here, using 13,198 genes, we have deduced the sets of genes involved in rate acceleration, positive selection, and relaxation of selective constraints in human, in chimp, and in their ancestral lineage since the divergence from murids. Significant deviations from the strict molecular clock were observed in 469 human and in 651 chimp genes. The more stringent branch-site test of positive selection detected 108 human and 577 chimp positively selected genes. An important proportion of the positively selected genes did not show a significant acceleration in rates, and similarly, many of the accelerated genes did not show significant signals of positive selection. Functional differentiation of genes under rate acceleration, positive selection, and relaxation was not statistically significant between human and chimp with the exception of terms related to *G-protein coupled receptors* and *sensory perception*. Both of these were over-represented under relaxation in human in relation to chimp. Comparing differences between derived and ancestral lineages, a more conspicuous change in trends seems to have favored positive selection in the human lineage. Since most of the positively selected genes are different under the same functional categories between these species, we suggest that the individual roles of the alternative positively selected genes may be an important factor underlying biological differences between these species.

## Synopsis

Since the publication of the human and the chimp genomes, one of the major challenges in evolutionary biology has begun to be deciphered: namely, the search for positively selected genes that have shaped humanness. Arbiza and colleagues undertake a genomic-scale search for the genes that have been positively selected in human, in chimp, and in their common ancestral lineage. They conclude that events of positive selection were six times more frequent in chimp than in human, although they do not group under specific functional classes that have been preferentially selected in either species. However, in the comparisons of the evolutionary trends between the ancestral and the descendant lineages, they found that most of the relative differences in common classes show an abundance of positive selection on the human branch. By differentiating positive selection from a relaxation of selective constraints, both producing analogous footprints in the genome, they demonstrate that many of the genes previously thought to have been positively selected correspond to likely cases of relaxation. Finally, they quantify the bias produced by the use of average rate–based approaches to concentrate cases of adaptive evolution in these species.

## Introduction

For years evolutionary biologists have been interested in knowing to what extent natural selection and genetic drift have shaped the genetic variation of populations and species [1–5]. Neutrality tests have provided powerful tools for developing hypotheses regarding this issue. The first objective of related studies had been to make general inferences about the causes of molecular evolution, and many efforts have been made to search for deviations from the molecular clock hypothesis. However, in the past ten years the focus has changed toward finding molecular events showing positive selection (PS) [6].

PS is the process favoring the retention in a population of those mutations that are beneficial to the reproductive success of individuals. Contrary to this process, the molecular clock hypothesis [7,8] postulates that the rate of evolution of molecular sequences is roughly constant over time. This observation has been taken as a strong evidence for the neutral mutation hypothesis [3], which postulates that the majority of molecular changes in evolution are due to neutral or nearly neutral mutations [2]. With the growing framework available for comparative genomic studies, it has been possible to test for neutrality against positive (or negative) selection at a genomic level.

Recent efforts at a large or genomic scale have been conducted to elucidate the intricacies of human evolution by means of comparing rate differences and PS against other fully sequenced species. In a recent work, Dorus et al. [9] found significantly higher rates of gene evolution in the primate nervous system when comparing against housekeeping and among subsets of brain-specific genes. From this data they proposed natural selection as the underlying mechanism. Other efforts have focused on finding direct molecular evidence of PS. Clark et al. [10], using more than 7,600 homologous sequences, found 1,547 human and 1,534 chimp genes as likely candidates to have been acted upon by PS. In a later study, Nielsen et al. [11], using more than 13,000 orthologous sequences, found that 733 genes deviated from strict neutrality, showing evidences of PS. In the latest genomic study published as of the time of this writing, the Chimpanzee Sequencing and Analysis Consortium (CSAC) found 585 out of 13,454 human–chimp orthologous genes as potential candidates to have been acted upon by PS, showing a Ka/Ki > 1 [12].

Indeed, while these three publications have been hallmarks in the genomic-scale search for events showing PS and have provided much insight into the subject, the combination of methods used have produced certain disagreements and have left some important considerations unaccounted for. As noted in the CSAC publication, the set of 585 genes observed may only be enriched for cases of PS given that, for example, the Ka/Ki statistic used could be >1 by chance in almost half of these genes if purifying selection is allowed to act non uniformly [12]. In Clark et al. [10], the branch-site test used for PS allowed distinguishing of lineage-specific cases of selection in the branches of human and of chimp, which has been criticized by other authors given that it may have suffered from the inclusion of false positives originating from the lack of power of the test to distinguish true cases of PS from cases of relaxation of selective constraints (RSC) [12,13]. The study by Nielsen et al. [11], with the exception of a small subset of 50 analyzed genes, was based on pair-wise comparisons that make it impossible to know in which of these lineages selection has occurred. In addition, in all of these studies, differentiation of the sets of genes under PS from the sets that are likely cases of RSC has not been done nor used specifically for study.

Finally, it is important to note that likelihood ratio tests like those used here and in some previous studies are sensitive to model assumptions [13,14]. While the tests used in this study have been shown to have a good performance under a variety of conditions [14], we prefer to address the definition of a genomic set of genes under PS from a conservative standpoint. Thus, while some of these studies have considered multiple testing corrections only for case-specific observations after comparisons, we have taken the approach of employing corrections for multiple testing as the norm for all comparisons, while considering the uncorrected sets for confirmation of specific results where appropriate.

Therefore many important questions regarding the identity and functional roles of genes showing acceleration, RSC, and PS, still remain: which are the genes that can be assigned to these sets with a considerable degree of sensitivity and confidence? Are these genes significantly different between species in functional terms? Do these genes encompass a special group of functional classes, or are they an unbiased representation of the genome? To what extent do the set of positively selected genes (PSG) differ from the set of accelerated genes? How many of the PSG can be distinguished from cases of RSC? Furthermore, can we gain any additional insight by comparing the pattern of adaptation of the derived species against that in their ancestral lineage?

All of these questions can only be answered by testing for deviations from the neutral theory in human, in chimp, and in their common ancestor, independently, using sensitive tests for PS while correcting for multiple testing. In this study, we have searched for the most complete set of known human genes with the chimp, mouse, rat, and dog orthologs available in order to answer all of these questions.

The two branch-site maximum likelihood (ML) tests of PS employed in this paper benefit from a high degree of sensitivity when compared with previous branch tests, and can be used together, as has been recently shown [14], in an approach that allows detecting lineage-specific events while distinguishing true cases of PS from likely cases of RSC. Both these tests are based on the comparison of the likelihood with which two alternative models fit sequence data. Test I compares the nearly neutral null model (M1a) against the alternative PS model (A). M1a assumes two codon site classes evolving under purifying selection and neutral evolution in all the lineages of the phylogeny. Model A considers two additional site classes conserved or evolving neutrally on all the branches (background lineages), except on a specified branch where PS is tested for (the foreground lineage). Test II compares the null model (A1) against the alternative model A. Parameters in model A1 are equal to those of model A with the exception that the two additional site classes in the foreground are only allowed to evolve neutrally. As was demonstrated by Zhang et al. [14], Test I cannot suitably distinguish cases of RSC from true events of PS, while Test II is able to make this distinction. One can therefore compare between the results of both tests in order to distinguish cases of PS from likely cases of RSC.

This is the first comparative genomic study where the lineage-specific events involved in processes of PS and RSC occurring in the human genome before and after the speciation event that differentiated us from our closest living species have been deduced.

## Results

### Testing the Molecular Clock Hypothesis

#### Relative rates test.

The analysis begins with the complete set of 30,709 genes in the Ensembl Human Database version 30.35c. These were filtered to remove all genes that had not been confirmed through mapping to Swiss-Prot, RefSeq, or SPTreEMBL, and a total of 20,469 genes, which in this manner had acquired the Ensembl known gene status, remained. Inspection of ortholog annotations for this set of genes in the Ensembl-Compara database (version 30) yielded 14,185 human genes with ortholog predictions in chimp, mouse, rat, and dog, corresponding to 69% of the known Ensembl human genome. After filtering the sequences by length and exceedingly high evolutionary rates, 13,197 genes were analysed by means of the relative rates test (RRT) (see Table S1). Evolutionary differences in rates between human and chimp were evaluated using Ka and Ks rates (Ka-RRT, Ks-RRT). Rate saturation was observed for 959 (7.3%) genes. After the RRT analysis, significant deviations from the molecular clock were observed for 844 (6.4%) human genes and for 1,260 (9.5%) chimp genes. After correcting for multiple testing (*p* < 0.05), the number of genes retained for further statistical analysis were 469 in human and 651 in chimp.

A more detailed analysis showed significant deviations in both Ka and Ks tests for 65 (0.5%) genes, out of which 18 evolved relatively faster in human than in chimp (HF), and 47 evolved relatively faster in chimp than in human (ChF). It is important to note that HF and ChF terms represent relative, rather than absolute, rate definitions. The number of genes for which there were significant differences, in either only Ka or only Ks, was higher for chimp (477 and 99) than for human (352 and 83), respectively. The RRT performed showed that a higher number of genes have significantly accelerated in nonsynonymous (938) rather than in synonymous changes (247). The ratio of the number of genes showing an acceleration of nonsynonymous to synonymous rates was similar and more than threefold (approximately 3.8) in both species. This bias constitutes an indirect evidence of the already characterized overdispersed clock in mammals, which suggests that protein evolution cannot be explained by a simple model theory of neutral evolution [1,15].

#### Rate differences in genes and species.

Table 1 shows the mean values obtained from RRT in the group of genes with significant deviations from the molecular clock hypothesis. They are arranged according to mutational changes (Ka and Ks), three ranges of *p*-values adjusted for multiple testing, and the two alternative directions of acceleration (HF or ChF).

**Table 1:**
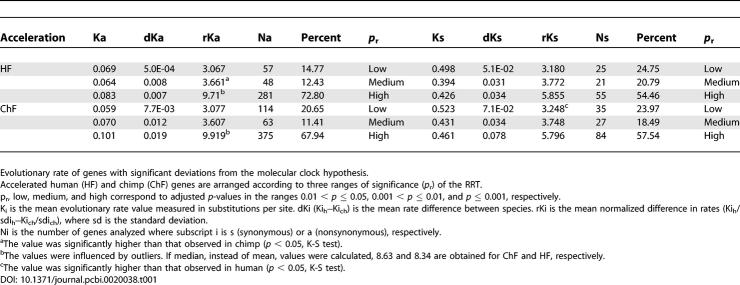
RRT Results

The bulk of all genes fall within the category showing the highest rates of evolution changing by nonsynonymous mutations (*p* < 0.001, *p*_r_ = high in Table 1), suggesting a favorable scenario for the presence of PS in human and in chimp. The Kolmogorov–Smirnov (K–S) test performed on mean normalized differences in rates (rKi in Table 1) detected significant differences in the medium rKa category, favoring human, and in the low rKs category, favoring chimp (*p* < 0.05). These minor differences were not sufficient to produce a net significant difference when comparing the full sets of genes without clock-like behavior in both species.

Table 2 shows the mean evolutionary rates estimated for human and for chimp using a topologically weighted outgroup, with mouse, rat, and dog as the reference in two alternative datasets. On the one hand, using only the group of genes showing significant RRT differences, the mean estimation of the human nonsynonymous rate of evolution (Ka = 0.079) was slower than that of chimp (Ka = 0.088), although the difference was not significant (*p* = 0.13). The same occurred for the synonymous rate change (*p* = 0.24). The relative evolutionary rate of chimp to human (R on Table 2) was 1.11 for Ka and 1.08 for Ks. On the other hand, when considering the full set of filtered orthologous genes, mean rates in substitutions per site were Ka = 0.086 and Ks = 0.430 for human, and Ka = 0.087 and Ks = 0.432 for chimp. Rate differences for Ka and Ks between species were again not significant. The mean Ka/Ks rate was similar between species and was slightly higher for the set of genes representing the complete genome than for those showing significant deviations from clock behavior (0.20 versus 0.18). This is due to the relative increase of the mean Ks rate observed on genes with significant deviations from clock (Table 2).

**Table 2:**
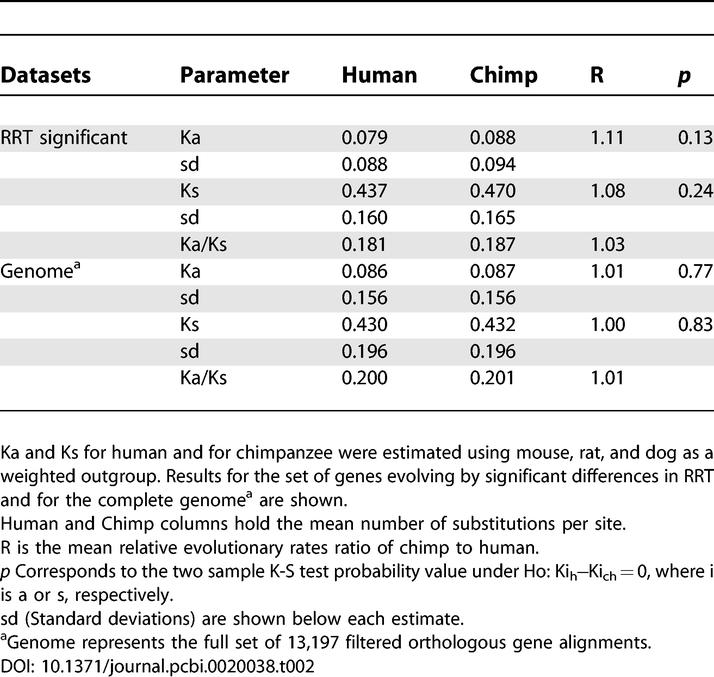
Evolutionary Rates of Human and of Chimp

ML estimations of evolutionary rates in the human branch and in the chimp branch were calculated using PAML [16] and compared with those recently obtained by the CSAC [12]. While our estimations were slightly faster for human (Ka = 0.0014, Ks = 0.0063 versus CSAC: Ka = 0.0013, Ks = 0.0062) and for chimp (Ka = 0.0015, Ks = 0.0066 versus CSAC: Ka = 0.0012, Ks = 0.0060), they were considerably similar to those obtained by the CSAC using a highly curated set of 7,043 orthologous genes [12]. The total number of genes with Ka/Ks > 1 was 445 in human and 539 in chimp, representing 5% and 6% of the total number of genes with a measurable ML estimation of the rates ratio, respectively.

#### Functional analysis of accelerated genes in human and in chimp.

Using human Gene Ontology (GO) terms [17], we have focused on seeing if there are any functional differences in the set of genes accelerated within the human genome and between both lineages. GO terms for chimpanzee were deduced from the corresponding human orthologs.

Table 3 shows the main GO terms corresponding to biological processes at GO level 6 associated to human and to chimp genes accelerated in synonymous and nonsynonymous changes. The most significant terms in the analysis of Ka and Ks are shown. The table is arranged according to those terms represented above 5% in the set of human nonsynonymous accelerated genes (column 1). Other terms above 5%, not shown in the table, were indeed observed in other categories (see Dataset S1 for a complete list of terms). For instance, *cation transport* (6.78%) was observed in the list of genes with coding sequences evolving faster in chimp than in human by means of nonsynonymous changes. Other terms such as *RNA metabolism, DNA metabolism, regulation of protein metabolism, regulation of programmed cell death, protein catabolism,* and *cellular carbohydrate metabolism* correspond to some of the human sequences and the chimp sequences accelerated by synonymous changes above 5%.

**Table 3:**
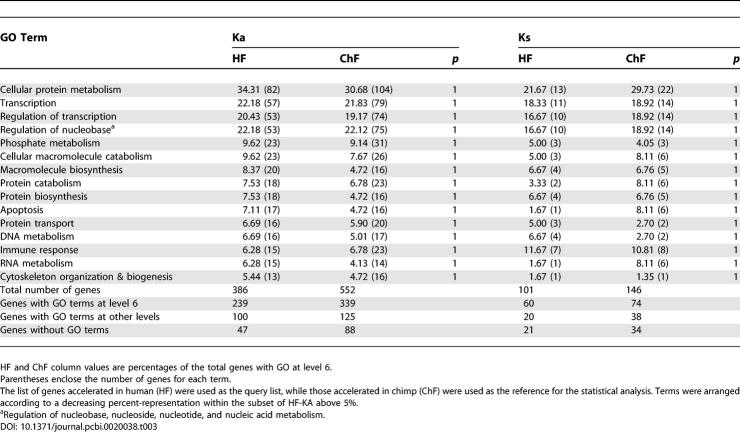
Functional Analysis of Genes with Deviations from the Molecular Clock

To find out if there were any over- or under-represented GO terms in between human and chimp, a Fisher exact test with *p*-values corrected for multiple testing was run using FatiGO [18,19]. Neither the test applied on HF and ChF genes with coding sequences evolving by means of nonsynonymous changes, nor that for synonymous ones, reported any significant difference for GO-term representation at any level (GO levels 3–6). We conclude that there are no statistically significant differences in functional GO classes represented in the sets of the genes without clock-like behavior between the two species. Finally, we tested the hypothesis that accelerated human genes represent an unbiased sample of the human genome in functional terms. Again, no GO terms were found to be significantly over- or under-represented among accelerated human genes when compared with the rest of the genome.

In summary, we have not detected GO terms differentially distributed between the significantly accelerated genes of human and of chimp. Moreover, the set of functions accelerated in human does not represent a special subset of genes with functional particularities within the human genome.

#### Testing adaptation in human and in chimp lineages.

The set of genes used for clock testing were also analyzed for signals of PS. After discarding those with fewer than three unique base pair differences, 9,674 human–chimp–mouse–rat–dog orthologous sequences remained. This set was then analyzed for signals of PS with Tests I and II, which can be used to distinguish RSC from true events of PS when used in conjunction with each other [14]. Both tests were performed on human and on chimp lineages, and 146 (1.51%) human and 672 (6.95%) chimp genes were obtained when the more restrictive Test II was considered. After correcting for multiple testing (*p* < 0.05), 108 (1.12%) and 577 (5.96%) genes in human and in chimp remained and were considered as true cases of PS occurring in their respective genomes.

#### Functional analysis of PSG.

Table 4 shows the main GO terms associated to the set of PSG detected using Test II in human and in chimp, as well as the difference in representation of GO terms for the sets of genes under PS for both species when compared with their ancestral lineage (see Dataset S1 for a complete list of terms). As before, terms shown are those represented above 5% in human PSG (H-PSG).

**Table 4:**
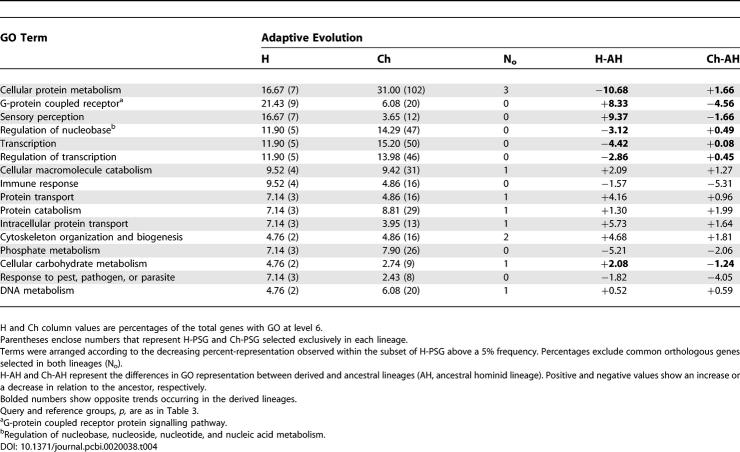
Functional Analysis of PSG

Initially, when comparing representations of terms under human and chimp directly, it is evident that with minor modifications of frequencies H-PSG have shown almost the same set of biological functions as those in chimp (Ch-PSG). It is interesting to note that in this comparison the highest differences in representation of genes between both lineages are found under terms such as *G-protein coupled receptor* (GPCR), *sensory perception, electron transport, integrin-mediated signalling pathway, inflammatory response,* and *cellular protein metabolism,* among others. All of these terms were represented to a greater extent in human with the exception of *cellular protein metabolism,* which was higher in chimp. Although the highest differences range from 4% to 15%, they were nonsignificant at any level (GO levels 3–6). Likewise, no term was significantly over- or under-represented in the comparison of H-PSG against the rest of the human genome. However, it is important to note that at least one difference seems evident: only a minor number of orthologous PSG are common between both species (N_o_ column in Table 4). This shows that PS-driven evolution of different genes under the same functional classes is the most frequent pattern occurring after speciation.

A more striking difference becomes noticeable when switching from the perspective of a direct comparison of the functional GO categories under PS for human and for chimp, to that based on the relative differences observed between the ancestral lineage and each one of the corresponding derived species. The H-AH and Ch-AH columns in Table 4 show the difference in representation of GO categories between the derived and ancestral lineages for human and for chimp, respectively. The representation of PSG under *G-protein coupled receptor, sensory perception,* and *cellular carbohydrate metabolism*, increase (+ values) in the human lineage while decreasing (− values) in chimp when compared with the ancestral lineage. In a similar but opposite manner, terms such as *cellular protein metabolism, transcription* and its regulation, *regulation of nucleobase, nucleoside, and nucleotide metabolism,* and *cellular carbohydrate metabolism* show a relative increase in chimp while decreasing in human. From this perspective, we can observe differences that could not be discerned from a direct comparison between derived lineages only: some terms have increased or decreased in relation to the ancestor in both species, others have changed in opposite directions in human and in chimp. The greatest relative differences observed (>10% between H-AH and Ch-AH) in the distribution of functional categories under PS correspond only to three categories: *cellular protein metabolism* which was comparatively favored by natural selection in chimp, and *G-coupled protein receptor signalling pathway* and *sensory perception,* comparatively favored in human. Finally, the relative differences observed in the remaining GO categories in Table 4 were below 5%.

### PS and Nonsynonymous Rate Acceleration

It is held that genes showing acceleration in nonsynonymous rate are likely to concentrate cases of PS. However, the comparison of Tables 3 and 4 reveals an outstanding difference between most of the represented GO categories under both processes. While four of the GO categories, each containing more than 50 genes with a significant nonsynonymous rate acceleration (Table 3), are within those most highly represented under PS in both species (Table 4), the terms *G-coupled protein receptor signalling pathway* and *sensory perception* were absent among those showing a significant acceleration in nonsynonymous rates. To understand these and other major discrepancies in the number of positives observed in Ka rate–based approaches and Test II, the relationship between the nonsynonymous rates difference (dKa = Ka_h_−Ka_ch_), the mean normalized differences in nonsynonymous rates between the species (rKa = dKa/sd), and the normalized nonsynonymous rate (Ka/Ks), were studied.

**Figure 1:**
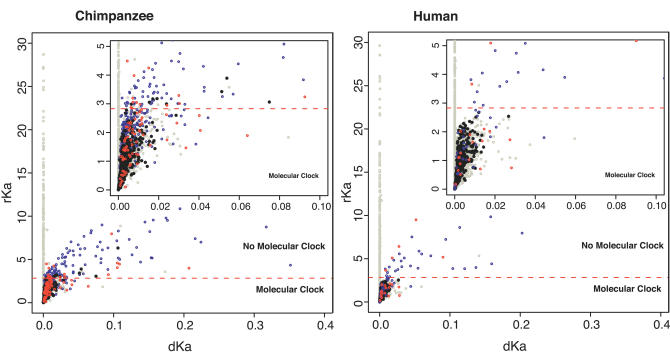
PS and Rates of Evolution A minor proportion of genes with Ka/Ks > 1 match events of PS in human and in chimp (red circles). Many of the genes with Ka/Ks < 1 show evidence of PS (blue circles). Genes with Ka/Ks > 1 without evidence of PS (black asterisks) fall mostly under molecular clock conditions for nonsynonymous changes (circles below the broken red line). Most of the genes without evidence of PS and Ka/Ks < 1 (grey circles) are scattered below the boundary limiting molecular clock like behavior and are observed at dKa < 0.0006 when molecular clock conditions are not fulfilled. Genes outside of clock conditions and dKa > 0.0006 coincide mostly with events of PS in both of the species (red and blue circles above the broken line). dKa and rKa as defined in Table 1.

Figure 1 shows the distribution of rKa versus dKa values for those genes with significant and nonsignificant differences in Ka-RRT (“molecular clock” in Figure 1). Under this distribution, four alternative groups have been labeled: those showing 1) both PS and Ka/Ks > 1 (red circles), 2) PS and Ka/Ks < 1 (blue circles), 3) Ka/Ks > 1 with no evidence of PS (black asterisks), and 4) Ka/Ks < 1 with no evidence of PS (grey circles).

The total number of genes with Ka/Ks > 1 considered in the analysis of Figure 1 was 336 in human (437 in chimp), out of which 22 (86) have shown evidence of PS (red circles in Figure 1) and only five (18) have shown significant deviations from the molecular clock in Ka rate (circles above the broken line). Alternatively, 58 human (407 chimp) genes with Ka/Ks < 1 were positively selected (blue circles). This shows that 72% of positively selected human genes did not show a Ka/Ks > 1 (82% in chimp). Similarly, 314 (93%) human and 351 (80%) chimp genes showing Ka/Ks > 1 have not shown evidences of PS (black asterisks). Notice that most of these genes have evolved without signs of nonsynonymous deviations from clock behaviour, suggesting that these values of Ka/Ks > 1 correspond to variations falling under a neutral model of evolution. The fact that many genes showed evidence of PS under clock-like behaviour (red and blue circles below the broken line) points out the high sensibility of the branch-site test employed where a few amino acid sites are probably involved in events of PS, without major changes in evolutionary rates between lineages (dKa).

In a similar manner, when considering differences in Ka rate instead of Ka/Ks rate ratios, 386 human genes (552 in chimp) have experienced a significant acceleration of nonsynonymous rate, and only approximately 32 of these genes (120 in chimp) have shown a reliable signal of PS. However, when considering genes with a significant acceleration in Ka rate and a dKa > 0.0006, most of them show evidence of PS (81% in human and 94% in chimp). Although it is important to remember that they are still a minority out of all of the genes with a significant deviation in Ka-RRT.

In summary, we observe that only those genes with a significant Ka-RRT and dKa > 0.0006 could possibly be considered as candidates for an enriched probability of having been positively selected. These results serve to highlight one of the downfalls of using elevated normalized Ka rates as a means of concentrating likely cases of PS in an a priori fashion.

### Ancestral and Derived Trends of RSC and PS

It is known that most tests of PS are not able to distinguish real events of positive Darwinian selection from cases of RSC [13]. This is the case with Test I used in this study. As has been previously demonstrated by Zang et al. [14], the genes observed exclusively in Test I but not in Test II correspond to likely cases of RSC.

**Figure 2:**
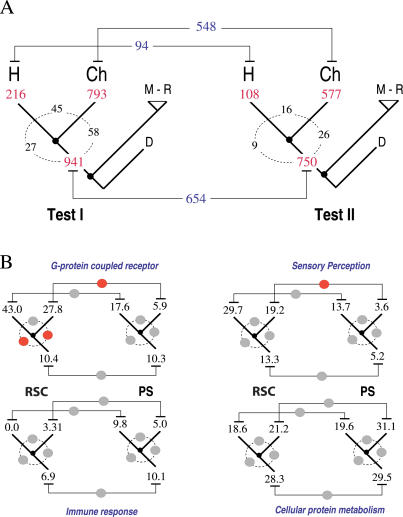
Phylogenetic Distribution of PSG under Tests I and II (A) The differential distribution of genes along tree branches, suggests a different pattern of occurrence of PS (Test II) and RSC (Test I) in derived and ancestral lineages. Numbers in red represent the total number of genes detected in each test after correcting for multiple testing. Numbers in black are common orthologous genes observed between lineages. Numbers in blue are genes observed in both tests. (B) The phylogenetic distribution of four representative GO categories is shown in human, in chimp, and in the ancestral lineage as depicted in the tree defined above. Numbers correspond to the percentage representation of genes under PS and RSC for each term out of the total number of genes with GO annotation. Filled circles show significant (red) and nonsignificant (grey) differences in the comparisons (see text for a detailed explanation).

Figure 2A shows the distribution of total and common genes observed in both tests for the three lineages analyzed. As expected, the great majority of H-PSG and Ch-PSG shown in Test II were also observed in Test I. After correcting for multiple testing, 216, 793, and 941 genes were detected in Test I for human, for chimp, and for the ancestral lineage, respectively. Only 122 human (1.26%), 245 chimp (2.53%), and 287 ancestral (2.97%) genes were found exclusively in Test I. This exclusive set of genes was used to study the functional classes associated to likely cases of RSC.

Figure 2B shows the results of the statistical comparisons performed (filled circles) between the representations of genes (numbers on branches) observed under PS and RSC between human, chimp, and the ancestral lineage for four functional GO categories. These categories were among those most represented within both tests, and serve at the same time as examples of the different patterns of differentiation observed between common categories of human and of chimp.

A common pattern observed for all of the functional categories represented in the set of genes under RSC was the absence of functional differentiation between human and chimp (grey-filled circles). However, a highly significant increase (red-filled circles) occurred in the representation of the term *G-protein coupled receptor protein signalling pathway* in the derived lineages in comparison with the ancestral lineage (Figure 2B). This significant over-representation of genes under RSC was higher for human (+32.68%, *p* < 1e-05) than for chimp (+18.36%, *p* = 0.006). Considering the time elapsed in each of the branches (approximately 75 Ma in the ancestral lineage against 5 Ma in the evolution of hominids), this suggests that a higher number of genes per unit time have experienced RSC after speciation in both this category and that of *sensory perception* (Figure 2B). Given that the relative representations of PSG belonging to *G-protein coupled receptor* and *sensory perception* increased in humans while decreasing in chimp after speciation (Table 4, Figure 2B), it is not surprising that statistically significant differences were only detected in chimp (red-filled circles). Furthermore, *G-protein coupled receptor* and *sensory perception* were statistically over-represented (*p* < 1e-05) when comparing the set of genes under RSC against the rest of the genes available in our dataset as representatives of the human genome. In summary, although both categories have increased in representation in human after speciation, a more frequent process of RSC has occurred under both of these, in both species.

The opposite pattern was observed for the *cellular protein metabolism* category (Figure 2B). In this case, the representation of genes under RSC decreased after speciation in both species. However, a higher representation of PSG under this category occurs in chimp and is the consequence of a marginal increase relative to the ancestral condition. A more pronounced reduction in the number of genes found under RSC occurred for the *immune response* category. In this case, no genes were observed to be under RSC in human, and considering the relative representation in each lineage, it seems to suggest that human showed little variation and chimp decreased in comparison to the ancestral proportion of PSG, while both species decreased under RSC.

**Figure 3:**
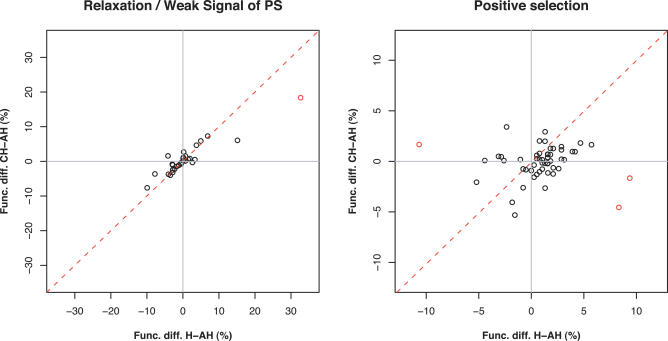
Ancestral and Derived Trends in Adaptation and RSC Differences in GO term representation between the sets of the derived and the ancestral lineages (H-AH, human versus ancestral lineage; CH-AH, chimp versus ancestral lineage) are plotted against each other using genes exclusively observed in Test I (RSC) and Test II (PS). Each quadrant represents a particular evolutionary scenario increasing or decreasing in GO representation for each of the lineages after speciation. Terms showing a difference in representation between H-AH and CH-AH >10% were labeled in red: *G-coupled protein receptor* was found in both Test I (14.32%) and Test II (12.89%), and *sensory perception* (11.03%) and *cellular protein metabolism* (−12.34%) in Test II. Only the terms common to all lineages are shown.

Figure 3 shows the evolutionary changes in representations before and after the speciation process for all of the common GO classes deduced under both tests. The difference in representation between human and the ancestral lineage for each functional term (H-AH) is plotted against the difference observed between chimp and the ancestral lineage (CH-AH). Each point represents a functional category, and depending on its location in each one of the quadrants (Q) under both graphs, alternative evolutionary scenarios can be deduced. The diagonal represents a homogeneous increase (positive values) or decrease (negative values) in relation to values observed for the ancestral lineage during the evolution of both species.

GO terms with positive differences in representation in both axes correspond to those increasing in both species after the speciation process (Q1). Considering the adaptive evolutionary process, a total of 26 functional categories fits this pattern (PS graph). Most of them (21) showed higher differences in representation in human than in chimp (H-AH%, Ch-AH%), i.e., *synaptic transmission* (1.57, 0.68), *detection of abiotic stimulus* (2.87, 0.21), *intracellular protein transport* (5.73, 1.64), *energy derivation by oxidation of organic components* (3.13, 0.16), and *small GTPase mediated signal transduction* (2.87, 1.14), among others. Another 20 GO terms showed a relative increase in their relative representation in human while decreasing in chimp after speciation (Q4), i.e., *G-protein coupled receptor* and *sensory perception* (differences in Table 4 and Figure 2B), *electron transport* (1.3, −2.65), *male gamete generation* (0.26, −1.57), *blood vessel morphogenesis* (1.04, −0.77) and *wound healing* (1.56, −0.23), among others. The opposite process, favoring the relative increase of PSG in chimp while decreasing in human, was detected for seven GO terms (Q3): *apoptosis* (−2.61, 0.07), *transcription* (−4.42, 0.08), *regulation of transcription* (−2.68, 0.45), and *cellular protein metabolism* (differences in Table 4 and Figure 2B), among others. Finally, a relative decrease from the ancestral representation of PSG was observed in six GO categories for both species (Q3): *inflammatory response* (−0.78, −2.61), *response to pest, pathogens, and parasites* (−1.82, −4.05), and *immune response* (differences in Table 4 and Figure 2B), among others.

In summary, although Test II detected a higher number of PSG in chimp than in human, and GO term representations between them were not significant, the comparison between ancestral and derived adaptive trends show that out of a total of 59 common GO terms to all lineages, 41 showed a higher proportion of PS events occurring in the human lineage. Only 11 terms showed a higher proportion of PSG in chimp. Additionally, the difference in data distributions between the sets of RSC/weak signal of PS and that of PS, suggested by Figure 3, is persuasive. While differences in the percentage of GO terms are widely distributed between the species, variations in GO representation of genes under RSC are highly correlated between variables (*p* = 3.6e-15) and fall mostly along the diagonal. The pattern describes a regular increase and decrease of genes undergoing RSC under each GO category at proportional and similar rates in both species after the speciation process. Only two of the GO terms deviated from this general pattern; *G-protein coupled receptor* and *sensory perception* were both located in Q1 below the diagonal, and serve to highlight the high proportion of genes under these categories that are likely cases of RSC in both species.

It is worth noting that the fact that many of the genes found exclusively in Test I have functionally important products, such as homeobox- and polymerase-related proteins among others, seems to suggest that it is highly improbable that all of them have undergone a process of RSC. Probably many of them are genes with a weak yet true signal of PS not sufficient to be detected by Test II (R. Nielsen, personal communication). It is evident that further statistical methods are necessary to accurately differentiate weak signals of PS from real cases of RSC.

### Functional Roles of PSG in Human and in Chimp

Table 5 shows the gene name of some of the PSG belonging to a select few of the more representative GO categories observed in the analyses. In agreement with the estimations based on an acceleration-rate approach [12], many of the selection events associated to sensory perception in human and in chimp were detected in different genes related to auditory perception. For instance, *EDN3* was positively selected in human and is related to sensorineural deafness and hypopigmentation [20]. *USH1* was positively selected in chimp, and its loss of function produces the most severe form of the Usher's syndrome [21]. However, PS on genes related to the perception of sound was also found in the ancestral lineage. For instance, the *KPTN* murine ortholog is a candidate gene for the Nijmegan waltzer mouse mutant, which has vestibular defects and a variable sensorineural hearing loss [22]. Other genes related to sensory perception were also found under PS: *taste perception* was principally observed in human and the ancestral lineage, *visual perception* and olfactory receptor genes were found in all of the lineages. Nevertheless, as was previously suggested [12,13], most of the events of RSC found under the *sensory perception* category involved olfactory receptors. RSC in olfactory receptors was abundant in all three lineages. One striking observation was the high number of genes related to visual perception under RSC in the ancestral lineage of hominids. Although further research on this group of genes would be required, the observation probably makes sense considering the functional change produced by the loss of the nocturnal way of life in higher primates [23].

**Table 5:**
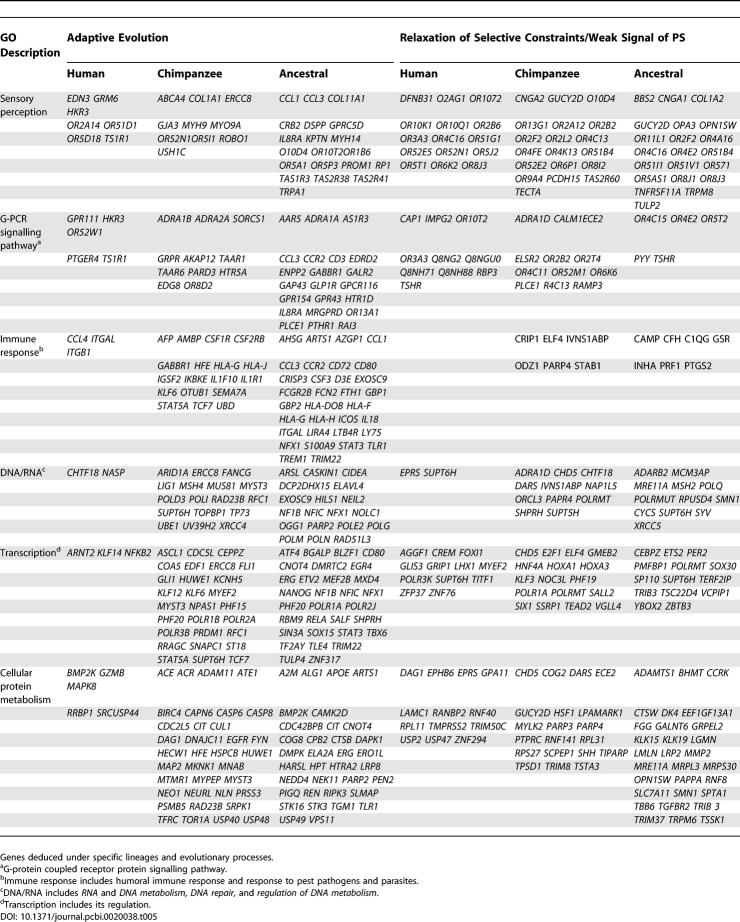
A Small Sample of the Human and the Chimp Genes Deduced under Tests I and II

Many other genes with a strong signal of PS in human (H), in chimp (Ch), in human and chimp (H-Ch), and in the ancestral lineage of hominids (AH) were related to: a) nervous system, H: *ARNT2* [24], H: *GFRalpha*-3 [25], Ch: *DRP2* [26], *NES* [27]; b) immune response, H: *PTGER4* [28], *CCL4* [29], Ch: *AFP* [30], *HLA-G* [31], H-Ch: *IGHG3* [32], AH: *HLA-DOB* [33]; c) cell cycle, H: *VEGFC* [34], Ch: *CCNE2* [35], AH: *EXT2* [36], *SEPTIN8* [37]; d) metabolism of xenobiotics, H: *ARNT2* [38]; Ch: *AKR1C1* [39], AH: *ABCB4* [40]; e) epidermis development, H: *KRA58* [41], Ch: *KRT10* [42], *COL7A1* [43], AH: *TGM5* [44], *KTR2A* [45]; f) inflammatory response, H: *ITGAL* [46], *CCL4* [29], Ch: *IL1F10* [47], *IL1R1* [48], AH: *CCL3, CCL1, CCR2* [49]; g) bone morphogenesis, H-CH-AH: BMP2K [50], Ch: *COL1A1* [51], *DCN* [52], AH: *BGLAP* [53], *AHSG* [54]; h) learning and memory, Ch: *FYN* [55], *GRIN2A* [56], AH: *APOE* [57] i) thyroid regulation, Ch: *SLC5A5* [58], *JMJD1C* [59]; AH: *CGA* [60], *PTHR1* [61]; and j) reproduction, Ch: *CGA* [62].

These functions are a small sample of those observed in this study and point out the great variety of functions modified by natural selection during hominid evolution.

## Discussion

We present a complete genomic evolutionary analysis of molecular clock, RSC, and PS considering the comparison with the ancestral lineage of hominids in order to differentiate adaptive trends in evolution after the speciation process differentiating human and chimpanzee. Based on testing deviations of neutrality in a gene-by-gene approach, we found a total of 1,182 (9.0%) human and 1,948 (14.8%) chimp genes with statistically significant deviations observed in at least one of the mentioned processes. However, after correcting for multiple testing we only considered 665 (5.0%) human and 1,341 (10.2%) chimp genes as a better estimate of the minimal sets under non-neutral evolution in these species. We conclude that these evolutionary processes do not show signs of being frequent events shaping the pattern of divergence between human and chimp genomes.

Differences in evolutionary rates exist between the species although there were no net significant differences. The number of genes showing a significant acceleration in non-synonymous rates exceeds those evolving by synonymous changes, and is greater for chimp than for humans. This excess of nonsynonymous changes favoring chimp correlates with the greater number of PS events observed in this species, and could be due in part to the comparatively smaller population size that has shaped human evolution [63].

For years, evolutionary biologists have known that deviations from the molecular clock, or rate acceleration in general, are not necessary, nor sufficient, to infer adaptive processes occurring during evolution of species. We have observed that a consideration of genes with a Ka/Ks > 1 yield a set where only 7%–20 % of genes show evidences of PS. Similarly, using a RRT approach on nonsynonymous mutations, those showing significant deviations are enriched for PS events from 10%–30%. With the addition of a nontrivial divergence value (dKa > 0.0006), the number of genes is reduced considerably, but PS events reach a concentration of 80%–95%. However, in all of these cases a high proportion of PSG are discarded in comparison with the number of PS events found by using the ML branch-site models of Test II used in this study.

A previous genomic study focusing on PS selection in human and in chimp has found that many functional categories were over- and under-represented in both species [10]. This was in disagreement with the results obtained in a posterior study [12] where only one GO category (developmental regulators) showed a possible over-representation in human in relation to chimp. In this publication, the possibility that the results of Clark et al. [10] were either likely to contain false positives involved in RSC or had RSC and PS correlated, was proposed. Our results tend to agree more with this last study, providing evidence for the lack of differentiation in functional classes of PSG in human and in chimp. Our results also support the notion that Clark's results may have included cases of RSC given that the model 2 test used in that study is very similar to Test I used here and that many of the deduced classes are here observed with a marked presence under RSC *(G-protein coupled receptor* and *sensory perception)*. However, a probable correlation between PS and RSC could not be discarded since highly represented functional classes under one of the processes are also highly represented in the other.

The sets of genes deduced without correction for multiple testing in molecular clock and PS analyses produced similar results for most of the GO representation comparisons observed after correction. The only exception was the term *G-protein coupled receptor protein signalling pathway* found to be additionally over-represented in human in relation to chimp under PS (Test II, *p* = 0.005). As previously mentioned, after correction for multiple testing we have not found GO terms over- or under-represented between both species. However, if differences between human and chimp are considered as independent trends evolving from the ancestral condition, a certain pattern seems apparent—although ancestral and descendent differences were not statistically significant. That is, we observe that a relative increase of PSG occurred in human for 41 out of the 59 GO categories common to all of the lineages, while only 11 showed a relative increase in chimp even though PSG in human are six times less than those in chimp. Although further studies would be required, this might suggest that in at least common fuctional GO classes, human has grown further apart from the ancestral lineage than chimp has through adaptive evolution. Finally, since most of the PSG are different between these species, the individual roles of the alternative PSG found associated under the same functional categories may be an important factor underlying biological differences between human and chimp.

Whole-genome analyses of evolutionary properties were made without any a priori hypothesis about the resulting genes. Consequently, these types of analyses are exhaustive and, at the same time, conservative regarding individual results. The necessity of keeping the type I error rate at an acceptable level leads to an unavoidable increase in the rejection of true positive results [64]. Therefore, the complete sets of accelerated and PSG we have found can only be considered their respectively most significant parts. The rest of the genes belonging to these categories must be found either by using hypothesis-driven approaches, or by means of more sensitive methodologies. In this study, previously discussed examples of PS, such as *FOXP2* and *BRCA1,* did not show evidence of PS. This would suggest that further detailed work on these genes is required.

For years it has been thought that the availability of the chimpanzee genome sequence and its comparison to that of human would reveal some of the molecular bases underlying the observable differences and possibly provide clues to that which makes us human. Now it is evident that neither the methodologies existing nor the detail and quality of the available annotation on the genes have allowed for a conclusive answer. In the future, new methods and more detailed functional annotations will be necessary to properly clarify this relevant biological issue.

## Materials and Methods

Ortholog annotations for the subset of 20,469 “known” Ensembl human protein-coding genes within the full set (30,709 genes) of the Ensembl version 30.35h H. sapiens database [65] were retrieved from the Ensembl-Compara database version 30 [66]. Coding sequences (CDS) for the proteins represented by the largest transcript of each ortholog were retrieved from the Ensembl databases (Human: version 30.35c, Chimp: version 30.2, Mouse: version 30.33f, Rat: version 30.34, Dog: version 30.1b).

DNA CDS were aligned using ClustalW [67] and parameters by default with translated protein sequences as templates. Codons containing gaps were removed. Alignments smaller than 50 bp were excluded from the analysis. The upper limit for Ka and Ks rates considered were those of the human interferon *γ* (Ka = 3.06) and the relaxin protein (Ks = 6.39 substitutions per site per 10^9^ years), showing the highest rates in human [5]. Assuming the human–mouse and human–chimp differentiation times to be about 80 million and 5 million years, respectively [68], all the comparisons with orthologs showing Ks ≥ 1 and Ka ≥ 0.5 substitutions/site for the RRT estimates, and those showing Ks ≥ 0.032 and Ka ≥ 0.0152 substitutions/site for ML lineage estimates, were excluded from the analysis. The RRT was performed using Li's method [69] as implemented in the RRTree program [70]. Sequences of human and of chimp were tested for deviation from a molecular clock using mouse, rat, and dog as the outgroup. Weights for each species in the outgroup were determined according to the topological scheme ((mouse:1/4, rat:1/4), dog:1/2)) as implemented in RRTree. Ka and Ks estimations were made on the CDS alignments of the largest transcripts of genes showing differences in GC content of less than 10%. Only three genes showed a GC content difference greater than 10% and were excluded from the analysis. Differences in human and in chimp rates were assessed using the Kolmogorov–Smirnov two-sample test [71]. ML estimations of Ka and Ks were computed jointly under a branch model for each ortholog using CodeML.

PS was evaluated using two different branch-site model Tests (I and II) [14], implemented in the CodeML program of the PAML (3.15) package [16]. Branches in the phylogeny were defined a priori as foreground and background lineages. Under these models only the foreground lineage may contain events of PS. Human, chimp, and their ancestral lineage, derived from the common ancestor of mouse and rat, were tested independently as the foreground lineage. Sequences with fewer than three unique base pair differences in codons between human and chimp were removed for the analysis of PS.

In contrast to the statistical behavior of previous branch-site tests [13], Tests I and II, developed and tested by Zhang et al. [14] and employed at a genomic scale in this study, are improved methods of branch-site test models using an ML approach which has proved to be more successful with regard to differentiating PS from RSC [14]. Test I compares M1a against model A. M1a assumes two site classes, 0 < ω_o_ < 1 and ω_1_ = 1, fixed in all the lineages of the phylogenetic tree. Model A considers four classes of sites. Site class 0 includes codons conserved throughout the tree with 0 < ω_o_ < 1. Site class 1 includes codons evolving neutrally throughout the tree with ω_1_ = 1. Site classes 2a and 2b include codons conserved or evolving neutrally on the background branches, but which become under PS on the foreground branches with ω_2_ > 1. The proportion p_i_ of the site classes (p_0_,p_1_,p_2_,p_3_) and the mean value of ω_2_ are estimated from the data by ML methods. Test II compares the null model A1 against model A. Parameters in A1 are equal to those of A with the exception that site classes 2a and 2b are fixed in the foreground with ω_2_ = 1. As was demonstrated by simulations [14], Test I cannot suitably distinguish cases of RSC from true events of PS. On the other hand, Test II, by allowing selectively constrained sites in the background to become relaxed under the proportion of site classes with ω_2_ = 1 set in the foreground of A1, is able to make this distinction, having an acceptable false discovery rate. One can therefore compare the results of both tests to distinguish cases of PS from events of RSC. Since the compared models are nested, likelihood ratio tests were performed and 2Δ values were posteriorly transformed into exact *p-*values using the pchisq function of the R statistical package [72]. The chi-squared distribution with d.f. = 2 and d.f. = 1, which have been shown to be conservative under conditions of PS [14], were used to perform Tests I and II, respectively.

In all cases, unless otherwise stated, p statistics derived from clock and PS analysis were false discovery rate–adjusted for multiple testing using the method of Benjamini and Hochberg [73]. Functional characterization of accelerated and PSG was carried out by means of the FatiGO program for functional annotation using GO [18,19]. FatiGO implements an inclusive analysis, where levels correspond to those in the directed acyclic graphs hierarchy defining the relationship between GO terms [74] which is chosen for the analysis [18,19]. The program computes a Fisher's two-tail exact test in order to statistically define over- or under-represented terms in between two lists of genes considering *p*-values corrected for multiple testing (false discovery rate–independent adjustment) [75].

## Supporting Information

Dataset S1 GO Functional Analysis Results of RRT and PS Tests (173 KB ZIP)

Table S1 Variables Obtained for All the Orthologous Genes (1.8 MB ZIP)

## Abbreviations

AH-PSG: PSG in the ancestral hominid lineage
CDS: coding sequences
ChF: chimp faster than human
Ch-PSG: chimp PSG
CSAC: Chimpanzee Sequencing and Analysis Consortium
GO: gene ontology
HF: human faster than chimp
H-PSG: human PSG
Ka: nonsynonymous rate of evolution in substitutions per site
Ka-RRT: relative rates test on nonsynonymous sites
K–S: Kolmogorov–Smirnov
Ks: synonymous rate of evolution in substitutions per site
ML: maximum-likelihood
PS: positive selection
PSG: positively selected genes
Q: quadrant
RRT: relative rates test
RSC: relaxation of selective constraints
